# Spatiotemporal Variation and Influencing Factors of TSP and Anions in Coastal Atmosphere of Zhanjiang City, China

**DOI:** 10.3390/ijerph19042030

**Published:** 2022-02-11

**Authors:** Ji-Biao Zhang, Yu-Mei Rong, Qi-Feng Yin, Peng Zhang, Li-Rong Zhao, Chun-Liang Chen

**Affiliations:** 1 College of Chemistry and Environmental Science, Guangdong Ocean University, Zhanjiang 524088, China; zhangjb@gdou.edu.cn (J.-B.Z.); rongyumei@nuit.edu.cn (Y.-M.R.); yinqf@mail.sustech.edu.cn (Q.-F.Y.); zhaolr@gdou.edu.cn (L.-R.Z.); 2 Southern Laboratory of Ocean Science and Engineering (Guangdong Zhanjiang), Zhanjiang 524088, China; 3 Analytical and Testing Centre, Guangdong Ocean University, Zhanjiang 524088, China; chencl89@gdou.edu.cn

**Keywords:** coastal atmosphere, anions, TSP, influencing factor, Zhanjiang

## Abstract

Water-soluble anions and suspended fine particles have negative impacts on ecosystems and human health, which is a current research hotspot. In this study, coastal suburb, coastal urban area, coastal tourist area, and coastal industrial area were explored to study the spatiotemporal variation and influencing factors of water-soluble anions and total suspended particles (TSP) in Zhanjiang atmosphere. In addition, on-site monitoring, laboratory testing, and analysis were used to identify the difference of each pollutant component at the sampling stations. The results showed that the average concentrations of Cl^−^, NO_3_^−^, SO_4_^2−^, PO_4_^3−^, and TSP were 29.8 μg/m^3^, 19.6 μg/m^3^, 45.6 μg/m^3^, 13.5 μg/m^3^, and 0.28 mg/m^3^, respectively. The concentration of Cl^−^, NO_3_^−^, PO_4_^3−^, and atmospheric TSP were the highest in coastal urban area, while the concentration of SO_4_^2−^ was the highest in coastal industrial area. Moreover, there were significantly seasonal differences in the concentration of various pollutants (*p* < 0.05). Cl^−^ and SO_4_^2−^ were high in summer, and NO_3_^−^ and TSP were high in winter. Cl^−^, SO_4_^2−^, PO_4_^3−^, and TSP had significant correlations with meteorological elements (temperature, relative humidity, atmospheric pressure, and wind speed). Besides, the results showed the areas with the most serious air pollution were coastal urban area and coastal industrial area. Moreover, the exhaust emissions from vehicles, urban enterprise emissions, and seawater evaporation were responsible for the serious air pollution in coastal urban area. It provided baseline information for the coastal atmospheric environment quality in Zhanjiang coastal city, which was critical to the mitigation strategies for the emission sources of air pollutants in the future.

## 1. Introduction

After decades of rapid economic development in China, people’s living standards have been generally improved, and demand for improving air quality is getting higher and higher. The main substances affecting air quality are coarse particulate matter and aerosol particles contained in the atmosphere. Atmospheric aerosols are gas dispersion systems in which fine particles, such as solids and liquids in the atmosphere, are dispersed in a gas and contain various substances [1]. Soluble anions (chloride ion, nitrate ion, phosphate ion, sulfate ion) and suspended fine particles are important environmental factors affecting atmospheric environmental quality. Atmospheric anions are converted mainly from their gaseous precursors, such as HCl, NO_X_, HNO_3_, HNO_2_, SO_2_, and liquid nitric acid mist and sulfuric acid mist. Previous studies have shown that the accumulation of atmospheric sulfate, nitrate, and PM_10_ levels can cause a variety of diseases, seriously affecting human health [2,3,4,5,6,7,8]. In recent years, researches on air pollutants focused on the temporal and spatial distribution characteristics, sources, and impact mechanisms of PM_2.5_, SO_2_, NO_2_, NH_3_, H_2_S, HNO_3_, and O_3_ in the atmosphere worldwide [9,10,11]. Some studies have shown that the distribution characteristics of atmospheric pollutants are closely related to meteorological elements and are affected by seasonal temperature, relative humidity, atmospheric pressure, wind direction, and wind speed. Scholars have studied the sources of various air pollutants in different research areas and consider that the sources of sulfur oxides and nitrogen oxides are industrial waste gas and biomass (coal, petroleum, etc.) combustion [12,13,14,15], while the gas conversion process of particles, road dust, and the main emissions combustion of biomass are the main sources of atmosphere pollutants [16,17,18,19,20,21,22,23,24,25,26].

Zhanjiang is a typical tropical ocean climate city, and the concentration levels and sources of air pollutants have not been thoroughly studied. Zhanjiang city is located at the southernmost tip of China mainland also in the southwestern of Guangdong Province and adjacent to the Tropic of Cancer in the north. Tropical and subtropical monsoon climates meet here and are regulated by the marine climate throughout the year [27]. Zhanjiang has no severe cold in winter and scorching heat in summer. According to statistics, the number of permanent residents in Zhanjiang in the past three years has been increasing yearly, at 8.30 million in 2016, 8.34 million in 2017, and 8.85 million in 2018. Moreover, Zhanjiang has vigorously developed its industry in recent years. Baosteel Zhanjiang Iron and Steel Co., Ltd. (Zhanjiang, China) officially started the construction at the Donghai Island of Zhanjiang on 31 May 2012 and was officially put into operation on 15 July 2016. In February 2019, BASF, a large German chemical company, officially announced the establishment of a new integrated base of fine chemical products on the Donghai Island of Zhanjiang. Due to the increase in the number of people and the large amount of pollutants caused by the introduction of chemical companies, the quality of atmospheric environment in Zhanjiang will undergo serious changes. At present, relatively few studies have assessed the levels and sources of air pollutants in Zhanjiang city.

Therefore, in this work, seasonal monitoring of atmospheric pollutants and total suspended particles (TSP) was carried out at four sites in coastal city, coastal urban area, coastal tourist area, and coastal industrial area. Atmospheric pollutant samples were collected by solution absorption method and analyzed by ion chromatography. The aims of this research were (1) to identify the spatiotemporal TSP variation in coastal city of Zhanjiang; (2) to explore spatiotemporal variation of atmospheric anions in coastal city Zhanjiang; and (3) to clarity the relationships among the TSP, atmospheric anions, and meteorological factors in Zhanjiang city. This study can provide a baseline scientific information for improving the coastal atmospheric environment quality in Zhanjiang city.

## 2. Materials and Methods 

### 2.1. Sampling Site and Time Schedule 

**Figure 1:**
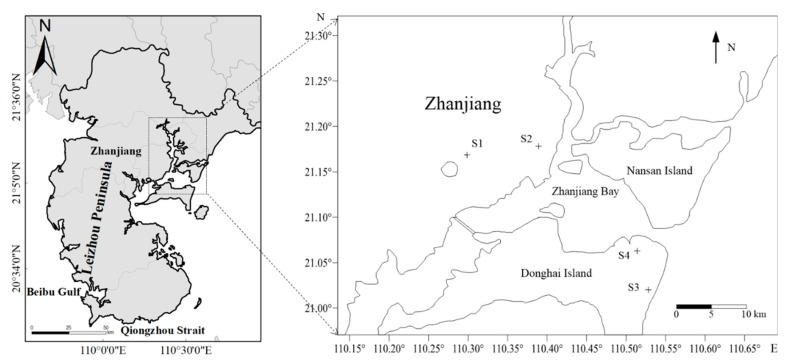
Location of the monitoring sites in Zhanjiang city.

Four sampling sites were selected in Zhanjiang to collect atmospheric pollutants and TSP samples: the main campus of Guangdong Ocean University (S1), the Xiashan campus of Guangdong Ocean University (S2), the beach in Donghai Island (S3), and the steel base on Donghai Island (S4) (Figure 1). The latitude and longitude of each station are shown in Table 1. Among these stations, S1 is located in the coastal suburb, S2 is located in the coastal urban area, S3 is located in the coastal tourist area, and S4 is in the coastal industrial area. From June 2018 to May 2019, 8–10 days of sunny weather in the representative month of each season were selected to conduct 24-h continuous sampling at four survey stations. The 24-h continuous sampling is divided into 4 time periods (0:00–6:00 h, 6:00–12:00 h, 12:00–18:00 h, and 18:00–24:00 h) at each site for collection. A total of 228 atmospheric absorption samples and 228 TSP samples were collected.

### 2.2. Sampling Instruments and Methods

Two parallel air TSP integrated samplers (Laoying 2050), equipped with a dust sampler and two air absorption collectors, were used to collect simultaneously coastal atmospheric pollutants and TSP samples. For air-soluble anions, each sampler was placed with two samples of 10 mL atmospheric absorption liquid (5% hydrogen peroxide); one was used to collect anions in the atmosphere, and another was used to collect blank samples at a zero flow rate. During the sampling process, one dust sampler with the GFF filter of 0.3-μm aperture was used to collect TSP samples in the atmosphere, and the other was used to collect a blank sample at a zero flow rate.

During sample collection, the flow rate of 0.2 L/min was used to collect atmospheric anion samples and zero flow rate for blank samples; the flow rate of 100 L/min was used to collect TSP samples and zero flow rate for blank TSP samples; the sampling was set to 6 h. While the sampler was working, weather data (temperature, relative humidity, pressure, wind speed, wind direction) were recorded synchronously by the PH-II-C handheld weather station. The concentration of the samples obtained in this experiment had subtracted the blank value.

### 2.3. Sample Pretreatment and Testing

#### 2.3.1. Sample Pretreatment

Before sampling, 10 mL of 5% aqueous solution of hydrogen peroxide was prepared as an atmospheric absorption liquid. The 1μm glass fiber filter membrane was first placed in a blast drying oven and baked at 105 °C for 3 h, then removed into a desiccator, and cooled at room temperature for 24 h. The electronic analytical balance (METTLER TOLEDO) was used for multiple weightings until the absolute value of the difference between the last two masses did not exceed 0.5 mg, and the average of the last two masses was recorded. Then, the fiber filter membrane was placed in the filter cartridge for TSP sampling. 

#### 2.3.2. Sample Testing

##### Testing of TSP Concentration

The filter with the TSP sample was weighed, and then, the mass concentration of TSP was obtained by the following formula.


$$C=(W_{1}-W_{0})\times 10^{3}/V_{S}$$

In the formula, *C* represents the mass concentration of TSP (mg/m^3^), *W*_0_ represents the mass (g) of the filter before sampling, *W*_1_ represents the mass (g) of the filter after sampling, and vs. represents the sampling volume (m^3^) under the standard condition (273.15 K, 101.325 kPa).

##### Testing of Anion Concentration in Atmospheric Absorption Liquid

The atmospheric absorption liquid sample was ultrasonically shaken for 1 h so that the water-soluble ions adsorbed on the particulate matter were sufficiently dissolved in water. Then, the sample was filtered through a 0.45-μm micro-porous membrane filter and finally analyzed by ion chromatograph.

Anion chromatograph (863 compact IC plus anion, Metrohm Co. Ltd., Herisau, Switzerland) was used to determine the ion concentration of atmospheric absorption liquid samples. The mobile phase was the mixed eluent of 0.0032 mol/L Na_2_CO_3_ and 0.001 mol/L NaHCO_3_. The injection volume was 10 μL for each injection.

### 2.4. Data Analysis and Statistical Methods

OriginLab software was used to draw the change curve of monitoring data. The map of sampling stations was graphed by ArcGIS and Surfer software 8. One-way analysis of variance (ANOVA) was used to examine the statistical differences in data between two or more groups. Correlation analysis between variables was determined by Pearson correlation between the environmental factors and TSP and anions using SPSS software. A probability level of 0.05 was used to determine significance.

## 3. Results

### 3.1. Seasonal Meteorological Environments Variation in Zhanjiang 

**Figure 2:**
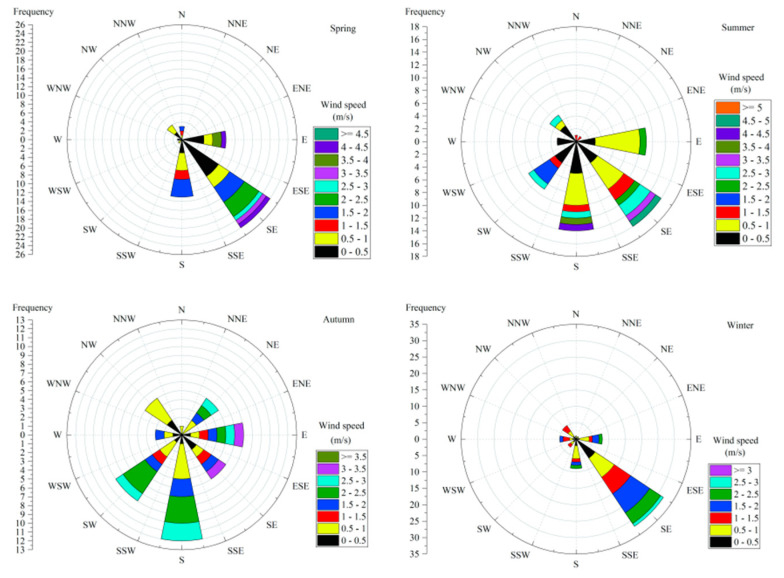
Wind rose diagram of Zhanjiang City from June 2018 to June 2019.

In this work, the basic meteorological environment of four monitoring stations in Zhanjiang was monitored for 114 days in four seasons from June 2018 to May 2019. For comparison purposes, the average data for each season were used to represent as the meteorological traits of Zhanjiang coastal atmosphere. The temperature in Zhanjiang atmosphere ranged from 16.5 °C to 39.1 °C, with an average of 25.2 °C. The summer temperature was the highest, with an average of 30.8 °C. The mean relative humidity was 82.0%, varying from 55.5% to 100%, and the average relative humidity was highest in spring at 86.1%. The average atmospheric pressure was 1008.6 hPa, varying from 989.1 to 1019.7 hPa, and the average atmospheric pressure was lowest in summer at 998.6 hPa (Table 2). Among these factors, wind direction and wind speed were represented by a rose diagram, which showed that in the four seasons, the southeast wind and the south wind were the dominant wind directions, and the wind speed was relatively strong in spring and summer (Figure 2).

### 3.2. Seasonal Air Pollutant Concentration Variation in Zhanjiang Atmosphere 

The average concentrations of coastal atmospheric pollutants of four seasons of each station are summarized in Table 3. The whole concentration levels of coastal atmospheric pollutants for four sampling stations were as follows: the coastal atmospheric chloride ion concentration ranged from 11.0 μg/m^3^ to 69.5 μg/m^3^ with the average of 29.8 μg/m^3^; the coastal atmospheric nitrate ion from 5.3 μg/m^3^ to 51.6 μg/m^3^ with the average of 19.6 μg/m^3^; the sulfate ion from 8.5 μg/m^3^ to 84.3 μg/m^3^ with the average of 45.6 μg/m^3^; the phosphate ion from 3.6 μg/m^3^ to 40.1 μg/m^3^ with the average of 13.5 μg/m^3^; and TSP from 0.03 μg/m^3^ to 0.59 mg/m^3^ and with the average of 0.28 mg/m^3^. There were obvious differences in the concentration of air pollutants at each survey station. The average concentrations of Cl^−^, NO_3_^−^, and TSP were the highest at S2 station; the average concentration of PO_4_^3−^ was the highest at S3 station; and the average concentration of SO_4_^2−^ was the highest at S4 station. In summary, the pollutant concentration of each station varied greatly, and the air pollutant concentrations were also different at different stations.

### 3.3. Coastal Atmospheric Pollutants Concentrations Variation with Meteorological Factors 

#### 3.3.1. Coastal Atmospheric Pollutants Concentrations Variation Affected by Temperature 

**Figure 3:**
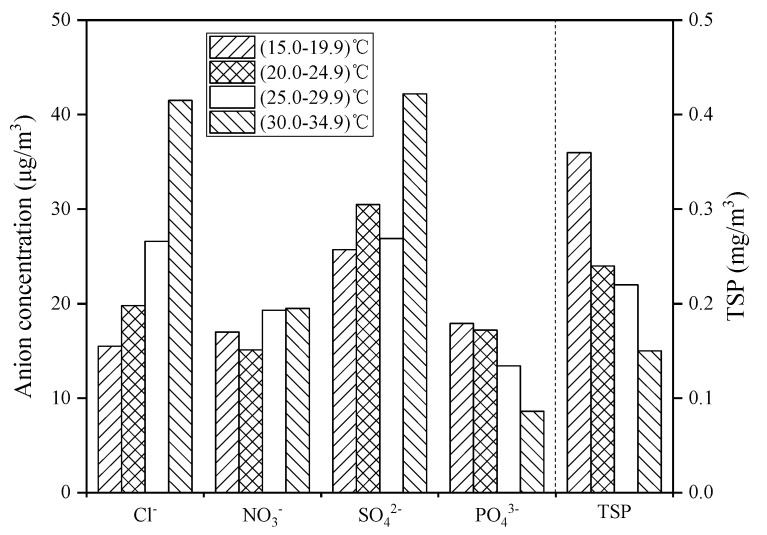
Relationship between temperature and atmospheric pollutant concentration.

During the sampling period, the highest, lowest, and average temperatures in Zhanjiang were 34.1 °C, 16.5 °C, and 25.2 °C, respectively, and for the sake of discussion, four temperature ranges were divided as 15.0–19.9 °C, 20.0–24.9 °C, 25.0–29.9 °C. and 30.0–34.9 °C, respectively. The changes of TSP concentration and coastal atmospheric anion concentrations with temperature during the entire sampling period are illustrated in Figure 3. In the temperature range of (30–34.9) °C, the concentrations of Cl^−^, NO_3_^−^, and SO_4_^2−^ were significantly higher, and the concentrations of PO_4_^3−^ and TSP were lower. With the increase of temperature, the concentration of Cl^−^, NO_3_^−^, and SO_4_^2−^ increased, while the concentration of PO_4_^3−^ and TSP decreased with the increase of temperature.

#### 3.3.2. Coastal Atmospheric Pollutants Concentrations Variation with Relative Humidity

**Figure 4:**
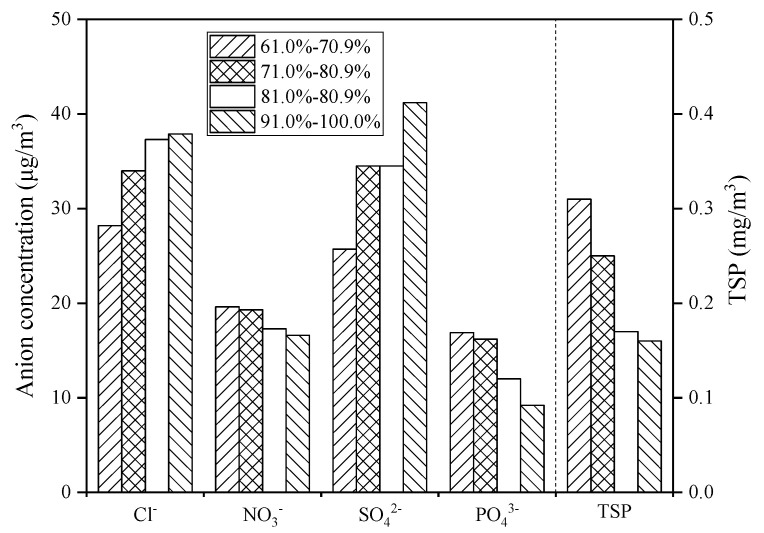
Relationship between relative humidity and atmospheric pollutant concentration.

During the sampling period, the highest, lowest, and average relative humidity in Zhanjiang atmosphere were 100%, 55.5%, and 82.0%, respectively, so the relative humidity is divided into four intervals for the convenience of analysis: 60.0–70.9%, 71.0–80.9%, 81.0–90.9%, and 91.0–100%. During the whole sampling period, with the increase of relative humidity, the concentration of Cl^−^ and SO_4_^2−^ increased, and the concentration of NO_3_^−^, PO_4_^3−^ and TSP decreased (Figure 4).

#### 3.3.3. Coastal Atmospheric Pollutants Concentrations Variation with Atmospheric Pressure

**Figure 5:**
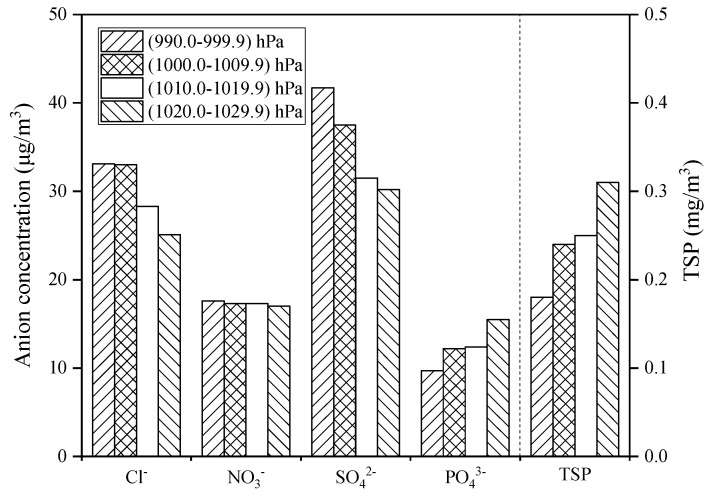
Relationship between atmospheric pressure and atmospheric pollutant concentration.

During the sampling period, the highest, lowest, and average atmospheric pressures in Zhanjiang were 1019.7 hPa, 989.1 hPa, and 1008.6 hPa, respectively. Additionally, for the convenience of analysis, the coastal atmospheric pressure is divided into four intervals: 990.0–999.9 hPa, 1000.0–1009.9 hPa, 1010.0–1019.9 hPa, and 1020.0–1029.9 hPa. During the entire sampling period, the concentrations of TSP and coastal atmospheric anions also changed with atmospheric pressure (Figure 5). It can be seen from the figure that with the increase of atmospheric pressure, the concentration of Cl^−^ and SO_4_^2−^ decreased, the concentration of PO_4_^3−^ and TSP increased, and the concentration of NO_3_^−^ varied very little with atmospheric pressure.

#### 3.3.4. Coastal Atmospheric Pollutants Concentrations Variation with Wind Speed and Direction

**Figure 6:**
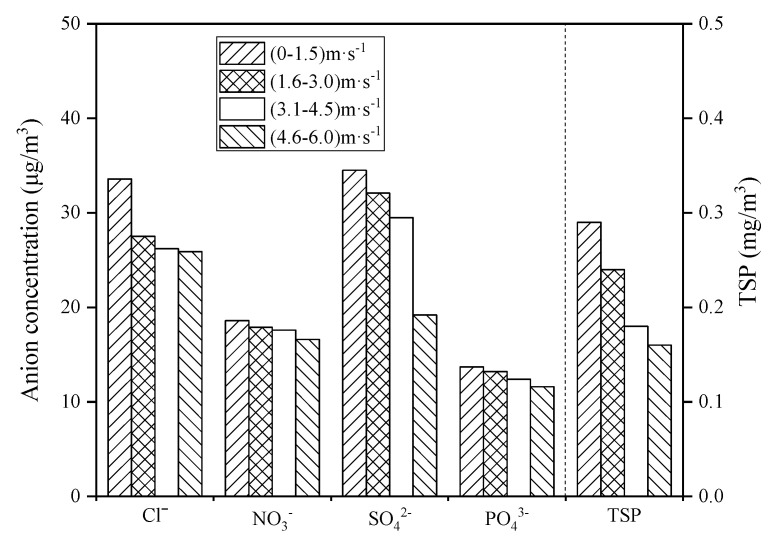
Relationship between wind speed and atmospheric pollutant concentration.

During the sampling period, the lowest, highest, and average wind speed in Zhanjiang was 0 m/s, 6.2 m/s, and 2.7 m/s, respectively. In this study, the wind speed was divided into four intervals: 0–1.5 m/s, 1.6–3.0 m/s, 3.1–4.5 m/s, and 4.6–6.0 m/s. The TSP and coastal atmospheric anion concentrations also varied with wind speed during the whole sampling period (Figure 6). As can be seen from the figure, the concentrations of each anion and TSP in the atmosphere decreased as the wind speed increased. It is noteworthy that when the wind speed was 4.6–6.0 m/s, the concentration of SO_4_^2−^ dropped sharply.

#### 3.3.5. Correlation Analysis of Coastal Atmospheric Anion Concentrations and Meteorological Factors

Using statistical analysis methods by SPSS software, we can quantitatively understand the relationship of coastal atmospheric anion concentration changes with various meteorological factors. The corresponding results of correlation analysis between atmospheric pollutants and meteorological factors are tabulated in Table 4. The result showed that the Cl^−^ concentration in the coastal atmosphere had a very significant positive correlation with air temperature and relative humidity and a negative correlation with atmospheric pressure and wind speed (Table 4). High temperature and relative humidity would promote high Cl^−^ concentration due to the significant positive correlations with each other. Atmospheric pressure and wind speed displayed a reverse contribution to the Cl^−^ concentration due to the negative correlations with each other. The correlation between NO_3_^−^ concentration and meteorological factors was not obvious, showing that NO_3_^−^ was less affected by meteorological factors. The concentration of SO_4_^2−^ was negatively correlated with atmospheric pressure and wind speed and had no significant correlation with temperature and relative humidity, showing that SO_4_^2−^ was less affected by temperature and relative humidity. The concentration of PO_4_^3−^ had a negative correlation with temperature and wind speed and a positive correlation with atmospheric pressure and relative humidity, showing that the concentration of PO_4_^3^^−^ displayed a decreasing trend with the increase of temperature and wind speed while displaying the synchronous changing trend as regards atmospheric pressure and relative humidity. The concentration of TSP displays a negative correlation with temperature, wind speed, and relative humidity but not atmosphere pressure. In summary, the concentration level and variation of atmospheric pollutants in Zhanjiang coastal atmosphere were influenced synthetically by multi-meteorological factors.

### 3.4. Seasonal Variation of Coastal Atmospheric Pollutants Concentration 

**Figure 7:**
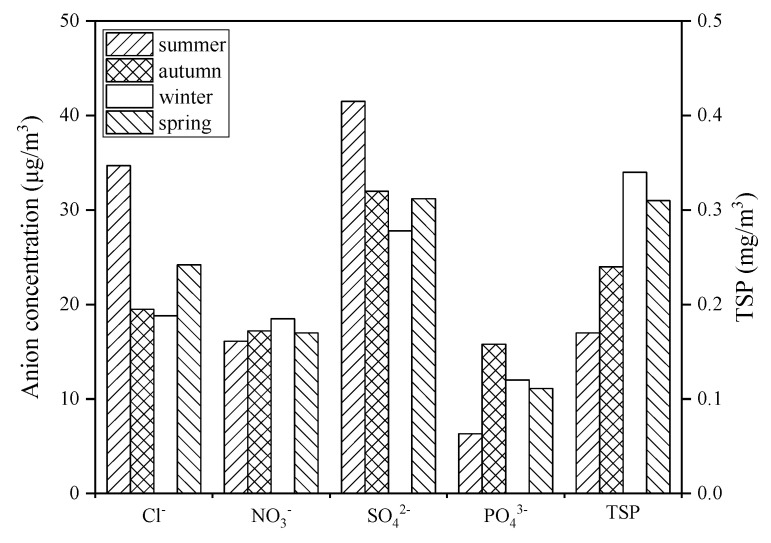
Atmospheric pollutant concentrations in different seasons.

According to the data analysis, it showed that when the season changed, the concentration of coastal atmospheric anions had obvious characteristics (Figure 7). The concentration of Cl^−^ and SO_4_^2−^ in the atmosphere was higher in summer and lower in winter. With the change of seasons, the difference in NO_3_^−^ concentration was not obvious. Meanwhile, the concentration of PO_4_^3−^ followed the sequence of autumn > winter > spring > summer, and the concentration of TSP followed the sequence of winter > spring > autumn > summer.

### 3.5. Changes in Atmospheric Pollutant Concentration in Different Sites

**Figure 8:**
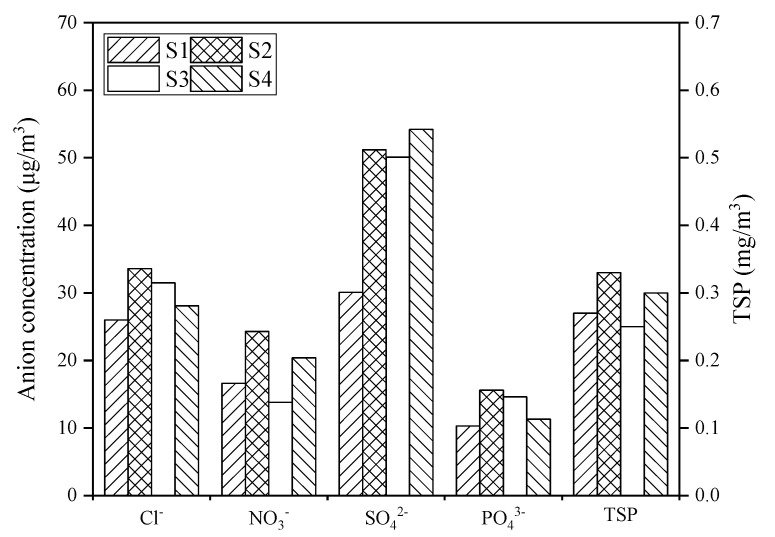
Concentration of atmospheric pollutants at different environmental areas (S1, coastal suburb; S2, coastal urban area; S3, coastal tourist area; S4, coastal industrial area).

The four sampling stations represented four typical types of coastal environments in Zhanjiang atmosphere, and their distances to the sea were also different (Table 1). For the different stations, the Cl^−^, NO_3_^−^, PO_4_^3−^, and coastal atmospheric TSP concentrations were the highest at S2 (coastal urban area), and the SO_4_^2−^ concentration at S4 (coastal industrial area) was the highest. The Cl^−^ and SO_4_^2−^ concentrations were the lowest at S1 (coastal suburb), and the NO_3_^−^, PO_4_^3−^, and TSP concentrations were the lowest at S3 (coastal tourist area). Overall, compared with other anions, the SO_4_^2−^ concentration was rather higher, and the concentration of various air pollutants varied significantly with the type of environment (Figure 8).

## 4. Discussion

### 4.1. Impact of Meteorological Factors on Coastal Atmospheric Pollutants Concentrations

Temperature has a significant effect on the changes of anion concentrations and TSP concentration in the coastal atmosphere (Figure 3). It may be because as the temperature increases, seawater will evaporate into the coastal atmosphere, and sea salt particles and further oxidized biogenic sulfide dimethyl sulfide (DMS) in the seawater will migrate into the atmosphere, resulting in high concentrations of Cl^−^ and SO_4_^2−^ in the tropical coastal atmosphere [28]. Automobile emissions are the main source of NO_3_^−^ [29]; therefore, the NO_3_^−^ concentration does not change significantly with temperature. The concentration of PO_4_^3−^ and TSP decreases with increasing temperature because PO_4_^3−^ and TSP are mainly land-based pollutants [30]. The high-temperature weather in Zhanjiang is often probably accompanied by rainfall. Under high-temperature and rainy conditions, the PO_4_^3−^ and TSP in the atmosphere settle down with the rain, so the PO_4_^3−^ and TSP decrease as the temperature rises. Zhanjiang city is located in a subtropical region and belongs to a subtropical monsoon climate with sufficient rainfall. Meanwhile, the ocean atmosphere along with the subtropical monsoon blows to the mainland, which brings a large amount of water vapor to ensure a high relative humidity in Zhanjiang atmosphere. With the increase of relative humidity, the land-derived pollutants NO_3_^−^, PO_4_^3^^−^, and TSP will partly settle with the condensation of water vapor, resulting in the decrease of the concentration of NO_3_^−^, PO_4_^3^^−^, and TSP in the atmosphere (Figure 4). The ocean atmosphere contains a large amount of water vapor, sea salt particles, and sulfate ions, which increase the concentration of Cl^−^ and SO_4_^2^^−^ in the coastal atmosphere [31]. In addition, when the atmospheric pressure increases (Figure 5), the air convection is not strong, and the rainfall is less, so the path of the Cl^−^ and SO_4_^2−^ transported through the ocean atmosphere is blocked, resulting in an overall decrease in the concentration of Cl^−^ and SO_4_^2−^, and with the decrease of tropical high-pressure rainfall, the accumulation of PO_4_^3−^ and TSP in the atmosphere has an increasing trend. Regarding wind speed, studies showed that strong wind could help to remove most of air pollutants in the coastal atmosphere [32]. Combined with the analysis of air pollutant concentration and wind speed in Zhanjiang (Figure 6), it shows that when the wind speed increases, the concentration of air pollutants and TSP both are reduced, which is consistent with the above research results.

### 4.2. Effects of Different Seasons on Coastal Atmospheric Pollutants Concentrations

Seasonal changes will affect the concentration of coastal atmosphere pollutants. The concentration of Cl^−^ and SO_4_^2^^−^ is higher in summer because of the dominant southeast wind and high temperature of Zhanjiang atmosphere in summer. A large number of sea salt particles evaporates from the surface seawater into the coastal atmosphere, and a large amount of SO_4_^2^^−^ and Cl^−^ are attached to the sea salt particles and are continuously transported to the terrestrial atmosphere with the southeast wind [33,34,35,36]. Moreover, the metabolism of seaweed and plankton metabolism also had a certain contribution to the SO_4_^2−^ concentration [37,38]. In Zhanjiang’s autumn and winter seasons, the dominant wind direction is northwest wind. Although a small amount of Cl^−^ and SO_4_^2−^ comes from exhaust emissions from factories and automobiles [39], Cl^−^ and SO_4_^2−^ mainly come from the ocean. Therefore, the concentration of Cl^−^ and SO_4_^2−^ in autumn and winter is lower than in summer due to a lower temperature. In addition, the concentration of NO_3_^−^ fluctuates slightly: the concentration in winter is slightly higher, and the concentration in summer is slightly lower. It may be that NO_3_^−^ mainly comes from the combustion of fuels in automobiles [40,41,42], factories [43] and other industries [44] as well as the combustion of trees, crops, and other plants [45,46]. These land-derived pollutants are blown from inland to the coast under the prevailing northwest wind in winter. Therefore, in the coastal atmosphere of Zhanjiang, the concentration of NO_3_^−^ in winter is higher than in other seasons. The concentration of PO_4_^3^^−^ is mainly affected by human activities, including the industrial production of various phosphate products, poultry excrement [47], solid waste [48], the use of phosphate fertilizers [49], and coal combustion [50]. The concentration of PO_4_^3−^ in autumn and winter is higher than that in spring and summer, mainly because there is more rainfall in spring and summer, which will make some of the PO_4_^3−^ in the atmosphere diminish. The concentration of TSP in the atmosphere is affected by natural and human factors. The natural influence is mainly from the accumulation of atmospheric particulate matter caused by wind and dust in the west and north direction [51]. Compared with natural sources of impact, human sources of impact are more complex, including industrial boilers [52], waste incineration [53,54], household heating [55], construction [56], and mining [57,58]. In general, TSP is mainly a land-based pollutant. The concentration of TSP in winter is higher than that in summer, mainly because the northwest wind prevails in winter, and there is more rainfall in summer. Notably, the atmospheric TSP concentration was slightly larger in spring than in autumn due to the increase of dewdrops in spring.

### 4.3. Impact of Different Environmental Types on Coastal Atmospheric Pollutants Concentrations

Based on the environmental characteristics of Zhanjiang coastal atmosphere, the S1 station (the main campus of Guangdong Ocean University) belongs to the typical coastal rural environment, so the concentrations of various anions and TSP are not high in this rural area [59]. Meanwhile, Cl^−^, SO_4_^2−^, and PO_4_^3−^ are the lowest among all investigated environmental types. Far from the sea, fewer types of enterprises and the small amount of motor vehicles are the main causes for the less polluted environment in this area. The S2 station (the Xiashan campus of Guangdong Ocean University) is a typical coastal urban environment, where the concentration level of anions and TSP in the atmosphere is the highest among all stations. Closer to the sea, a large number of motor vehicles and many urban enterprises (especially the catering industry) make a common contribution to the serious atmosphere pollution [60]. It means that fuel combustion, motor vehicle emissions, and the contributions from the ocean are the main sources of air pollution in the coastal urban atmosphere of Zhanjiang. The S3 station (the beach in Donghai Island), which is close to the area of steel industry, is a typical coastal atmospheric environment, so the pollutant concentration in this area was generally maintained at a medium level, and the main pollution cause was the contribution of evaporating from seawater and the diffusion effect of pollutants from the nearby steel industry atmosphere [61]. The S4 station (the steel base in Donghai Island) is a typical coastal industrial environment, so the concentration of various polluting anions and TSP in this area is relatively high. Its main sources of NO_x_ and SO_x_ are the contribution of evaporation from seawater and the industrial atmospheric emissions. However, due to the non-continuous and limited data at present, the different source contributions should be traced and explored by on-line monitoring and dynamic models in depth, which is critical to protect the Zhanjiang air quality under the great anthropogenic emission pressure in the future. 

## 5. Conclusions

There are various kinds of air pollutants that have an impact on air environmental quality. Soluble anions and total suspended particles are two important types of air pollutants. In this work, through the on-site monitoring in four typical environmental areas of Zhanjiang city atmosphere, the temporal and spatial distribution characteristics of water-soluble anions and TSP were obtained. The results indicated that the concentration changes of coastal atmospheric pollutants showed significant relationships with meteorological factors. The concentration of PO_4_^3−^ and TSP was negatively correlated with temperature, relative humidity, and air pressure. However, the concentrations of Cl^−^ and SO_4_^2−^ were positively correlated with those environmental factors. In addition, as the wind speed increased, the concentration of each pollutant decreased as a whole. Furthermore, sea wind helps to raise the concentration of soluble anions in atmosphere. Moreover, the concentrations of various coastal anions and TSP changed greatly in different seasons. During sampling periods, the concentrations of Cl^−^ and SO_4_^2−^ were largest in summer and lowest in winter. The differences in NO_3_^−^ concentration were not significant between seasonal changes. Comparatively, the concentrations of anions and TSP follow the order of coastal urban atmosphere > coastal industrial area ≈ coastal tourist area > coastal suburb in Zhanjiang coastal atmosphere. The contributions of motor vehicle emissions, urban enterprise emissions, and seawater evaporation were the main causes of air pollution in Zhanjiang coastal atmosphere. To avoid the deterioration decline of air quality, the mitigation strategies should be traced to the emission sources of air pollutants in the future.

## Figures and Tables

**Table 1:** Longitude and latitude of each station and the distance from sea.

Station	Longitude (N)	Latitude (E)	Distance from Sea (km)
S1	21°9′40.68″	110°17′43.44″	6.3
S2	21°11′8.52″	110°23′35.52″	0.9
S3	21°1′33.59″	110°31′32.52″	0.3
S4	21°1′33.95″	110°31′13.44″	2.0

**Table 2:** The statistics results of meteorological factors from June 2018 to June 2019.

Season	T(℃)	RH (%)	Air Pressure (hPa)
Spring	24.9	86.1	1009.8
Summer	30.8	79.6	998.6
Autumn	24.9	81.4	1011.7
Winter	19.6	81.2	1015.2
Maximum	34.1	100.0	1019.7
Minimum	16.5	55.5	989.1
Average	25.2	82.0	1008.6

**Table 3:** Seasonal air pollutant concentration in each station from June 2018 to June 2019.

Station	Range and Average	Cl^−^ (μg/m^3^)	NO_3_^−^ (μg/m^3^)	SO_4_^2−^ (μg/m^3^)	PO_4_^3−^ (μg/m^3^)	TSP (mg/m^3^)
S1	Maximum	53.5	29.7	52.0	31.2	0.45
	Minimum	12.1	5.3	8.5	5.0	0.08
	Average	26.4 ± 17.6	19.0 ± 5.2	29.6 ± 14.9	10.6 ± 13.6	0.26 ± 0.17
S2	Maximum	69.5	51.6	72.2	32.5	0.59
	Minimum	12.7	10.8	28.6	3.6	0.05
	Average	33.0 ± 19.5	25.1 ± 7.0	50.3 ± 13.7	15.1 ± 11.1	0.34 ± 0.22
S3	Maximum	60.8	24.8	70.6	29.0	0.58
	Minimum	11.0	7.6	15.0	8.1	0.03
	Average	30.5 ± 16.1	14.7 ± 4.6	49.1 ± 16.2	17.2 ± 10.7	0.25 ± 0.20
S4	Maximum	62.1	32.0	84.3	40.1	0.41
	Minimum	17.2	11.5	10.5	5.4	0.12
	Average	29.3 ± 16.5	19.5 ± 3.9	53.2 ± 18.1	11.0 ± 11.0	0.28 ± 0.19
Sum	Maximum	69.5	51.6	84.3	40.1	0.59
	Minimum	11.0	5.3	8.5	3.6	0.03
	Average	29.8 ± 11.1	19.6 ± 4.7	45.6 ± 16.1	13.5 ± 12.4	0.28 ± 0.18

**Table 4:** Pearson correlation between meteorological factors and atmospheric pollutant concentrations (*n* = 228).

Correlation Coefficient	Temperature (°C)	Relative Humidity (%)	Atmospheric Pressure (hPa)	Wind Speed (m·s^−1^)
Cl^−^	0.343 **	0.315 **	−0.238 *	−0.204 *
NO_3_^−^	0.027	−0.014	−0.010	−0.021
SO_4_^2−^	0.140	−0.197	−0.249 *	−0.266 *
PO_4_^3−^	−0.216 *	0.224 *	0.217 *	−0.202 *
TSP	−0.303 **	−0.224 *	0.291 *	−0.325 **

* Refers to the correlation is significant at *p* < 0.05 (two-tailed). ** Refers to the correlation is significant at *p* < 0.01.

## Data Availability

Data sharing is not applicable to this article.
